# Receptor-like cytoplasmic kinase ScRIPK in sugarcane regulates disease resistance and drought tolerance in *Arabidopsis*

**DOI:** 10.3389/fpls.2023.1191449

**Published:** 2023-05-25

**Authors:** Jinlan Fang, Zhe Chai, Run Huang, Cuilin Huang, Zhenhua Ming, Baoshan Chen, Wei Yao, Muqing Zhang

**Affiliations:** ^1^ College of Agricultural, Guangxi University, Nanning, China; ^2^ State Key Lab for Conservation and Utilization of Subtropical Agri-Biological Resources and Guangxi Key Lab for Sugarcane Biology, Guangxi University, Nanning, China

**Keywords:** sugarcane, kinases, ScRIPK, ScRIN4, drought, disease infection

## Abstract

**Introduction:**

Receptor-like cytoplastic kinases (RLCKs) are known in many plants to be involved in various processes of plant growth and development and regulate plant immunity to pathogen infection. Environmental stimuli such as pathogen infection and drought restrict the crop yield and interfere with plant growth. However, the function of RLCKs in sugarcane remains unclear.

**Methods and results:**

In this study, a member of the RLCK VII subfamily, ScRIPK, was identified in sugarcane based on sequence similarity to the rice and *Arabidopsis* RLCKs. ScRIPK was localized to the plasma membrane, as predicted, and the expression of *ScRIPK* was responsive to polyethylene glycol treatment and *Fusarium sacchari* infection. Overexpression of *ScRIPK* in *Arabidopsis* enhanced drought tolerance and disease susceptibility of seedlings. Moreover, the crystal structure of the ScRIPK kinase domain (ScRIPK KD) and the mutant proteins (ScRIPK-KD K124R and ScRIPK-KD S253A|T254A) were characterized in order to determine the activation mechanism. We also identified ScRIN4 as the interacting protein of ScRIPK.

**Discussion:**

Our work identified a RLCK in sugarcane, providing a potential target for sugarcane responses to disease infection and drought, and a structural basis for kinase activation mechanisms.

## 1 Introduction

Sugarcane (*Saccharum* spp. hybrids), the polyploid interspecific hybrids, is one of the primary sources of sugar production, and the significant energy material for ethanol production (Matsuoka et al., 2009). Plants live in constantly changing environments, which can be hostile and stressful for growth and development (Yamaguchi-Shinozaki and Shinozaki, 2006; Zhu, 2016). Pathogen infection significantly affects the sugarcane yield and juice quality, reducing their commercial use. Susceptible cultivars initially exhibit typical symptoms of white, narrow, and sharply defined leaf stripes, and eventually leaf wilt and complete necrosis, resulting in plant death (Rott et al., 1997; Rott et al., 2000; Rott et al., 2010). The cane yield and total dry matter of sugarcane are reduced by 17–52% and 20–56%, respectively, as a result of water limitation. (Basnayake et al., 2012).

The receptor-like protein kinases (RLKs) are some of the largest plant proteins, which are known to early recognize external signals and translate into cellular responses, which is composed of an extracellular domain, a transmembrane domain, and an intracellular domain (Shiu and Bleecker, 2003; Shiu et al., 2004). RLKs that lack an extracellular domain are called receptor-like cytoplastic kinases (RLCKs) (Shiu and Bleecker, 2001), and are predicted to be localized to the plasma membrane by post-translation N-terminal myristoylation motif (Maurer-Stroh et al., 2002). The plasma membrane localization enhances the interaction between RLCKs and other membrane proteins such as RLKs, forming receptor complexes and mediating growth, development, and immune responses in *Arabidopsis* and rice (Lin et al., 2013; Yamaguchi et al., 2013). Members of the *Arabidopsis* RLCK VII subfamily play important roles in pattern-triggered immunity. The *Arabidopsis* mutants *rlck vii*-*5*, *rlck vii*-*7*, and *rlck vii*-*8* have been shown to compromise the production of reactive oxygen species (ROS) in response to all patterns tested (Rao et al., 2018). The *Arabidopsis* RLCK BIK1 interacts with WRKY transcription factors involved in the jasmonic acid (JA) and salicylic acid (SA) regulation. The *Arabidopsis* RLCK *RIPK* knockout and overexpression lines exhibit enhanced disease resistance and susceptibility, respectively (Liu et al., 2011). A *Glycine soja* calcium-dependent calmodulin-binding receptor-like kinase (*GsCBRLK*) is induced by drought, salinity, and abscisic acid (ABA), and its overexpression in *Arabidopsis* enhances plant tolerance to high salinity and ABA (Yang et al., 2010). A rice RLCK, *BRORD-SPECTRUM RESISTANCE 1* (*OsBSR1*), is overexpressed in tomato, torenia, *Arabidopsis*, and sugarcane, and those *OsBSR1*-overexpressing transgenic plants exhibit resistance to bacteria and/or fungus (Maeda et al., 2023). The RLCK member AtCRK36 interacts with and phosphorylates AtARCK1 to form a complex and negatively control abiotic stress signal transduction (Tanaka et al., 2012). The rice OsRLCK253 interacts with A20/AN1 zinc-finger containing stress-associated protein OsSAP1/11 and confers drought and salt stress tolerance in *Arabidopsis* (Giri et al., 2011). The other rice drought-inducible RLCK, *GROWTH UNDER DROUGHT KINASE* (*GUDK*, *OsRIPK*), plays essential roles in grain yield under drought and natural conditions, and loss-of-function mutant lines display sensitivity to abiotic stresses (salinity stress and osmotic stress), and abscisic acid treatment at the seedling stage (Ramegowda et al., 2014).

RLCKs have been shown to work synergistically with receptor kinases or receptor-like proteins to regulate the plant’s innate immunity, cope with abiotic stresses and regulate other developmental processes, and RLCKs regulate receptor kinases-/receptor-like proteins-mediated signaling by phosphorylating various downstream components and modulating the activity of receptor complexes (Lu et al., 2010; Lin et al., 2013; Li et al., 2014; Yu et al., 2017). Phosphomimetic mutations of AtBIK1 S89 and T90 (EFR-phosphorylated sites) lead to increased phytohormones and enhanced resistance to bacterial infections (Lal et al., 2018). As a component of FLS2 immune receptor complex, AtBIK1 positively regulates calcium influx triggered by flg22 and directly phosphorylates NADPH oxidase RbohD at specific sites in a calcium independent manner to promote ROS production in *Arabidopsis* (Li et al., 2014). In response to bacterial effectors, AtRIPK interacts with and phosphorylates AtRIN4, whose phosphomimetic mutants show constitutive activation of RPM1-mediated defense responses (Liu et al., 2011). OsGUDK phosphorylates and activates OsAP37 to mediate drought stress signaling (Ramegowda et al., 2014). However, few studies have focused on individual sugarcane RLCKs, their functions in pathogen infection and abiotic stress, and the associated mechanism of autophosphorylation.

In this study, we aimed to identify a putative RLCK gene in sugarcane, analyze its function, and study its autophosphorylation mechanism. We identified ScRIPK, a member of the RLCK VII subfamily, by a screen for sugarcane orthologs of *Arabidopsis* RIPK and rice GUDK. Subcellular localization, RT-qPCR, and ectopic expression in *Arabidopsis* were used to analyze the localization and function of ScRIPK. In addition, the crystal structure of the ScRIPK kinase domain was determined to understand its conformation and autophosphorylation mechanism. This study isolated and analyzed the conserved phosphorylated sites of ScRIN4. Bimolecular fluorescence complementation and luciferase complementation imaging assays were used to determine the interaction between ScRIPK and ScRIN4. Our finding provides evidence for the function of *ScRIPK* in bacterial infection and drought in sugarcane and provides valuable insight into the regulation of kinase activity.

## 2 Materials and methods

### 2.1 Plant materials and growth conditions

Sugarcane cultivar ROC22 was cultured in the sugarcane clonal germplasm repository at Guangxi University (Nanning, China) and grown at 30°C in a greenhouse with a 13 h light/11 h dark cycle. *Arabidopsis* (Columbia, Col-0) plants were grown at 22°C under long-day conditions (16 h light/8 h dark), which provided the genetic background of plants overexpressing *ScRIPK*, whose coding region was cloned into the pBWA(V)HS vector, driven by 35s promoters. T_2_ seedlings were used for analysis. *Nicotiana benthamiana* (*N. benthamiana*) plants were grown in a growth chamber under long day conditions (16 h light/8 h dark) at 26°C, with a light intensity of 150 µmol m^–2^ s^–1^ and 70% relative humidity. Four-week-old *N. benthamiana* was used for bimolecular fluorescence complementation analysis and luciferase complementation imaging assays.

### 2.2 Treatments

The 1-month-old (5–7 leaf stage) sugarcane plants with uniform growth were irrigated once with 500 mL 250 mM NaCl and 20% (p/v) Polyethylene glycol (PEG) 6000 in the greenhouse, respectively (Nogueira et al., 2005; Chu et al., 2022). All leaves were then sampled at 0, 1, 3, 6, 12, 24, and 72 h after treatment. Ten sugarcane seedlings of per replicate were inoculated with *Fusarium sacchari* (1 × 10^5^ conidia ml^−1^) at the 5-leaf stage and +1 leaves were sampled after at 0, 12, 24, 72, 120, and 168 h post-inoculation, which performed as described (Huang et al., 2022; Paul et al., 2022). Three biological replicates were performed. All samples were collected and immediately frozen in liquid nitrogen, and stored at −80°C.

For the *Arabidopsis* experiments, transgenic and wild-type seeds were germinated on 1/2 Murashige and Skoog medium (1/2 MS) for 10 days under the same conditions as described in the section on plant materials and growth conditions. For the drought treatment, seedlings were transferred from 1/2 MS to soil, and then 4-week-old seedlings were not watered for 10 days. After recovery for 3 days, the relative water content (RWC) was measured as previously described (Zhang et al., 2019). For the *Pseudomonas syringae* pv. *tomato* (*Pst*) DC3000 infection experiment, *Pst* DC3000 was grown in King’s B broth (Coolaber, Beijing, China), and then diluted with 10 mM MgCl_2_ solution to OD_600_ 0.001. Leaves of 4-week-old transgenic plants and wild-type plants were infiltrated with *Pst* DC3000 using a syringe without a needle for 3 days.

### 2.3 Subcellular localization

The coding sequence of *ScRIPK* was amplified, and then inserted into pEarleyGate 103 vector, which performed as described previously (Fang et al., 2021; Chai et al., 2022). The pEarleyGate-103-ScRIPK vector was transiently expressed in sugarcane protoplasts with the plasma membrane marker vector. After 16–20 h, fluorescence was observed and imaged under a confocal scanning microscope (Leica-TCS-SP8MP; Leica Microsystems, USA). Three biological replicates were performed.

### 2.4 RNA extraction and RT-qPCR assays

Total RNA from sugarcane leaves and whole seedlings of *Arabidopsis* were extracted and purified using the Eastep Super Total RNA Extraction Kit (Promega, USA). The cDNA was synthesized with a PrimeScript RT reagent kit with gDNA Eraser (Takara, USA), and the cDNA was used for RT-qPCR assays, which performed as described previously (Fang et al., 2021; Chai et al., 2022). RT-qPCRs were performed with a procedure as follows: 95°C 30s for initial denaturation; 95°C 5s, 60°C 30s for denaturation, annealing and extension with 40 cycles; and 95°C 5s, 60°C 1min, 95°C 1s for melt curve analysis. The relative expression levels were calculated following the 2^-ΔΔCt^ method. *GADPH* (Coelho et al., 2014) and *Atactin2* (Shahnejat-Bushehri et al., 2016) were used as references to determine relative expression in sugarcane and *Arabidopsis*, respectively. Primers used in this study are provided in **Supplementary Table 1**.

### 2.5 Expression and purification of ScRIPK, ScRIPK K124R, and ScRIPK S253A|T254A

The kinase domain (72–378 aa) of ScRIPK and mutants (ScRIPK K124R and ScRIPK S253A|T254A) were amplified in 25 µL reaction mixture containing 1 µL of cDNA template, 2.5 µL of specific forward and reverse primers (2 µM), 2.5 mM dNTP mixture (2.5 mM), 12.5 µL of 2 × GC buffer II, 0.5 µL of Takara LA Taq (Takara, USA). Steps were as follows: after initial denaturation at 94°C for 4 min, 35 cycles were run at 94°C for 30 s, 60°C for 30 s, and 72°C for 1 min 30 s, then extension at 72°C for 10 min and stored at 4°C. The two products were separately cloned into the PRSF-duet vector with the In-Fusion HD Cloning Kit (50°C for 10 min, and then placed on ice, Takara, USA) and verified by sequencing (Sangon, China). These vectors were transformed into *Escherichia coli* strain BL21 (DE3, TransGen Biotech, China) for protein expression. Single clones were then picked and cultured in the Luria-Bertani (LB) medium at 37°C until OD_600_ reached 0.6–1.0. Then, 0.5 mM isopropyl-beta-D-thiogalactopyranoside (IPTG) was added to induce protein expression at 16°C for 18–20 h. Cells were centrifuged and then lysed with PBS (2.7 mM KCl, 10 mM Na_2_HPO_4_, 1.8 mM KH_2_PO_4_, and 10 mM MgCl_2_) buffer containing 500 mM NaCl, 20 mM imidazole, and 0.01% Tween 20, and centrifuged at 12,000 rpm for 1 h at 4°C. Soluble protein was then bound to the Ni-NTA column (affinity chromatography, Smart-Lifesciences, China) for preliminary purification and the target protein was eluted by the PBS buffer containing 300 mM imidazole, and 0.01% Tween 20, which was further removed non-target proteins by Superdex^TM^75 (gel filtration chromatography, GE Healthcare, USA) at 16°C with the buffer containing 20 mM Tris PH 7.4, 300 mM NaCl, 4 mM MgCl_2_, and 0.01% Tween 20.

### 2.6 Crystallization, data collection and structure determination

The ScRIPK-ATP, ScRIPK K124R-ATP, and ScRIPK S253A|T254A-ADP complex were Crystallized using the hanging-drop method by mixing 1 µl protein with equal volume of reservoir solution in 24-well plates at 291 K (Hampton Research, Aliso Viejo, CA). The ScRIPK-ATP protein complex crystal was successfully obtained in the reservoir solution containing 0.1 M HEPES pH 7.5, 40% v/v PEG 400. ScRIPK K124R-ATP and ScRIPK S253A|T254A-ADP protein complex crystals were successfully obtained in the reservoir solution containing 0.1 M HEPES pH 7.5, 4.3 M Sodium chloride. These crystal data were collected on beamline BL17U1 at the Shanghai Synchrotron Radiation Facility (SSRF) and processed by XDS. The three structures were determined by molecular replacement, using the structure of BIK1 (PDB ID: 5TOS) as the initial search model. Model building and structural refinement were analyzed using Coot and PHENIX, respectively. The refinement statistics are summarized in **Supplementary Table 1**. The atomic coordinates and structure data (ScRIPK-ATP, PDB ID: 8HO6; ScRIPK K124R-ATP, PDB ID: 8HOA; ScRIPK S253A|T254A-ADP, PDB ID: 8HOD) have been deposited in the Protein Data Bank. All structure figures were prepared using PyMOL.

### 2.7 *In vitro* kinase assays

For the kinase assays, 2 µg recombinant protein was run in the buffer (25 mM Tris-HCl PH 7.4, 10 mM MgCl_2_, 1 mM ATP, and 1 mM DTT) in a total volume of 25 µL at room temperature for 30 min. Then the reaction was terminated by adding 5× protein loading buffer and boiling for 5 min at 100°C. Proteins were then separated on a 12% SDS-PAGE gel, stained with Coomassie blue G250, or transferred to a nitrocellulose membrane (PALL, USA) for protein immunoblotting using anti-Phospho-(Ser/Thr) antibody (Abcam, UK).

### 2.8 Bimolecular fluorescence complementation

For BiFC analysis, the full-length sequence of *ScRIPK* was constructed into pEarleyGate201-YN (YN) and pEarleyGate201-YC (YC) and the full-length sequence of *ScRIN4* was also constructed into YN and YC. The YN-ScRIPK and YC-ScRIN4 plasmids, or YN-ScRIPK and YC-ScRIN4 plasmids, were then transformed into *N. benthamiana* leaves. The positive controls (YN-AtRIPK + YC-AtRIN4 and YN-AtRIN4 + YC-AtRIPK) and the negative controls (YN-ScRIPK + YC, YN + YC-ScRIPK, YN-ScRIN4 + YC, YN + YC-ScRIN4, YN-AtRIN4 + YC, YN + YC-AtRIN4, YN-AtRIPK + YC, YN + YC-AtRIPK, and YN + YC) were also transformed into *N. benthamiana* leaves. After 2 days, images were captured with a confocal scanning microscope (Leica-TCS-SP8MP, Leica Microsystems, USA). Three biological replicates were performed.

### 2.9 Luciferase complementation imaging assays

For luciferase complementation imaging assays (LCI), the full-length coding sequence of *ScRIPK*/*AtRIPK* was cloned into Nluc, and the full-length coding sequence of *ScRIN4*/*AtRIN4* was cloned into Cluc. The Nluc-ScRIPK, Cluc-ScRIN4, Nluc-AtRIPK, Cluc-AtRIN4, and the empty vectors Nluc and Cluc were transformed into *Agrobacterium* strain GV3101, respectively. Then, Nluc-ScRIPK was mixed with Cluc-ScRIN4 for the transient infiltration of *N. benthamiana* leaves. The same was done for Nluc-AtRIPK and Cluc-AtRIN4 as the positive control. Nluc-ScRIPK + Cluc, Nluc + Cluc-ScRIN4, Nluc-AtRIPK+ Cluc, Nluc + Cluc-AtRIN4, and Nluc + Cluc were used as the controls. After 2 days, LUC activity was observed and imaged using a CDD imaging apparatus (IVIS Lumina LT, PerkinElmer, USA).

### 2.10 Statistical analysis

Student’s *t*-test and Duncan’s multiple range test were performed in our study to determine their significance using the SPSS software (IBM Analytics, USA).

## 3 Result

### 3.1 Isolation and characterization of ScRIPK

**Figure 1:**
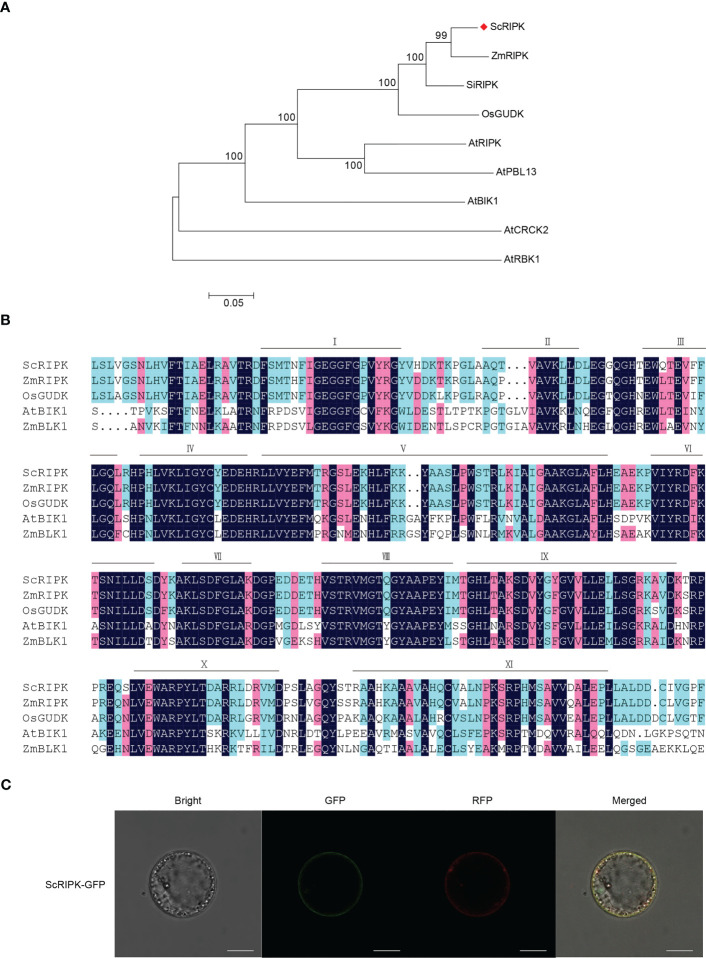
Characterization of ScRIPK. **(A)** Phylogenetic analysis of ScRIPK orthologs. The phylogenetic tree was generated with 9 full-length amino acid sequences of RLCKs using MEGA with the Neighbor-Joining method, 1,000 replicates, and p-distance method. **(B)** Amino acid sequence alignment of ScRIPK, ZmRIPK, OsGUDK, AtBIK1, and ZmBLK1 kinase domains. Bars indicate the eleven conserved subdomains (I–XI). **(C)** Subcellular localization of ScRIPK in sugarcane protoplasts. The vector pEarleyGate 103-ScRIPK was transformed in sugarcane protoplasts together with the plasma membrane vector fused with RFP. GFP represents green fluorescent protein, RFP represents red fluorescent protein. This experiment was replicated three times. Scale bar = 10 µm.

To explore the function of the *AtRIPK* and *OsGUDK* homologous genes in sugarcane, we cloned and sequenced a sequence from the sugarcane cultivar ROC22. The deduced coding sequence contains a 1278 bp open reading frame, encoding a 425 amino acid protein with a predicted molecular weight of 46.36 kDa. BLAST analysis revealed that the deduced protein shares high similarity to *Zea mays* ZmRIPK, *Setaria italica* SiRIPK and *Oryza sativa* Japonica OsGUDK (88.02%, 86.14% and 82.91% amino acid identity, respectively), which was in the same clade as RLCK class VII (RLCK VII, **Figure 1A**). Therefore, this gene was designated ScRIPK. Like other kinases, ScRIPK protein kinase domain (residues 95 to 369) has 11 major conserved subdomains, I to XI (**Figure 1B**). These conserved subdomains are necessary for the catalytic function.

AtRIPK and related RLCKs contain an N-terminal palmitoylation/myristoylation motif that should serve as a targeting signal for plasma membrane localization (Veronese et al., 2006; Liu et al., 2011). To confirm the predicted ScRIPK localization, we conducted subcellular localization experiments using sugarcane protoplasts, which indicated that ScRIPK localized in the plasma membrane (**Figure 1C**). Thus, ScRIPK is a member of the RLCK VII subfamily localized in the plasma membrane.

### 3.2 Transcription responses of *ScRIPK* during abiotic and biotic stress conditions

**Figure 2:**
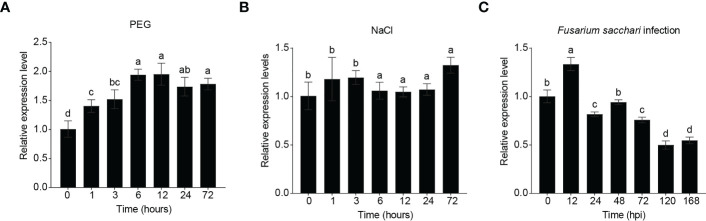
Relative expression of *ScRIPK* under different stress treatments. RNA was isolated from plants at the 5–7/5 leaf stage and with plant material sampled after treatment with polyethylene glycol (PEG, **(A)**), NaCl **(B)**, and *Fusarium sacchari* infection **(C)**. X-axis corresponds to the time point after each treatment application. The columns with the same letters are not significantly (*p*-value>0.05) different according to Duncan’s multiple range test. Error values are means ± SD, n = 5 (PEG, NaCl), n = 10 (*Fusarium sacchari* infection).

To test whether *ScRIPK* expression responds to abiotic and/or biotic stresses, we analyzed the transcript levels of *ScRIPK* under polyethylene glycol (PEG), NaCl, and pathogen infection conditions (**Figure 2**). *ScRIPK* transcription increased as early as 1 h after PEG treatment and exhibited an approximately 2-fold peak response at 6 h (**Figure 2A**). *ScRIPK* transcription levels had no noticeable change under NaCl treatment conditions (**Figure 2B**), but did respond to biotic stresses. *ScRIPK* transcription revealed a slight increase as early as 12 h post-fungus *Fusarium sacchari* treatment inoculation, followed by a downward trend until 168 h post-inoculation, with a total decrease of approximately 0.5-fold (**Figure 2C**). These data suggest that *ScRIPK* may play a role in responding to biotic and abiotic stresses.

### 3.3 Ectopic expression of *ScRIPK* enhanced drought tolerance and disease susceptibility

**Figure 3:**
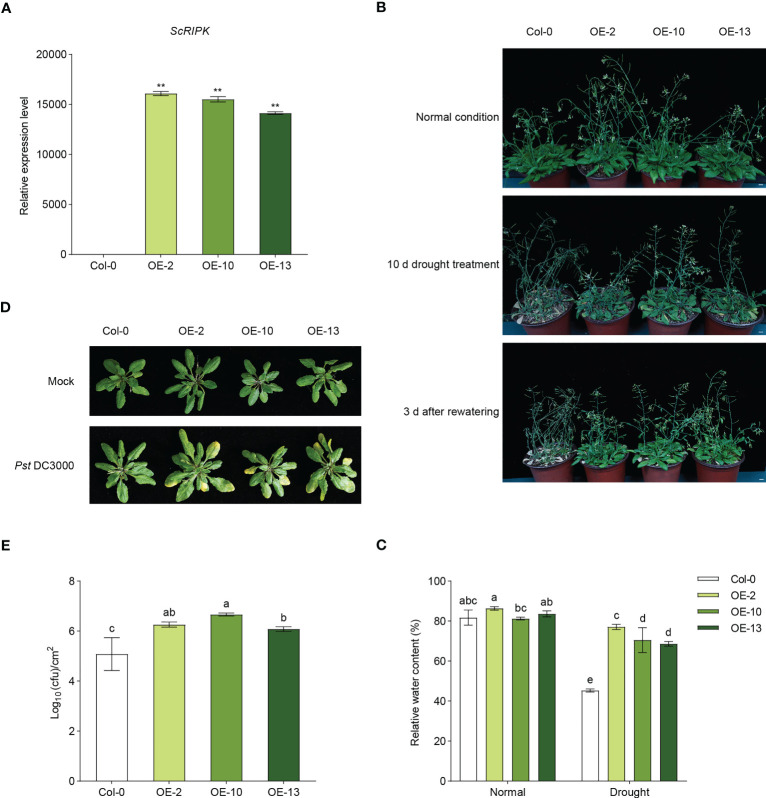
Overexpression of ScRIPK increased the drought tolerance and disease susceptibility in *Arabidopsis*. **(A)** RT-qPCR showed the expression of ScRIPK in *Arabidopsis* wild-type (Col-0) and overexpression (OE) lines. **p <0.01 compared with Col-0 using Student’s *t*-test. Error values are means ± SD, n= 3. **(B)** The four-week-old seedlings of Col-0 and OE lines were withheld for 10 d, followed by rewatering for 3 d Phenotype took at the normal condition, at the end of stress period (10 d) and 3 d after recovery. Bars = 1 cm. **(C)** The relative water content of wild-type (Col-0) and overexpressed (OE) lines after water or withholding water for 10 d The columns with the same letters are not significantly (*p*-value>0.05) different according to Duncan’s multiple range test. Error values are means ± SD, n = 5. **(D, E)** Four-week-old Col-0 and OE lines were syringe inoculated with *Pst* DC3000 (OD ≈ 0.001). Three days after inoculation, plants were subjected to photographed and growth analysis. N = 5. The columns with the same letters are not significantly (*p*-value>0.05) different according to Duncan’s multiple range test. Error values are means ± SD, n = 5.

To investigate the function of *ScRIPK*, we examined transgenic *Arabidopsis* plants over-expressing *ScRIPK*, driven by the constitutive 35s CaMV promoter. RT-qPCR was used to measure the expression levels of transgenic lines and three independent T_2_ lines (OE-2, OE-10, and OE-13) were subsequently selected for further analysis (**Figure 3A**). We performed a drought tolerance test in which 4-week-old seedlings of both wild-type lines and transgenic lines were not watered for 10 days and then rewatered for 3 days. After 10 days of withholding water, most of the leaves in transgenic plants were green in color and showed signs of slight water loss. In contrast, most wild-type plants’ leaves were yellow in color, rolled, and showed signs of severe water loss. Three days after rewatering, most of the leaves of the transgenic plants were completely restored and fully expanded. The leaves of wild-type plants did not recover and most of them were white (**Figures 3B, C**). To analyze the function of *ScRIPK* in plant biotic stress, wild-type plants and transgenic plants were inoculated with *Pseudomonas syringae* pv. *tomato* (*Pst*) DC3000 using syringe infiltration, and the progress of the infection was observed over 3 days. As shown in **Figure 3D**, wild-type plants did not exhibit macroscopic visible signs of infection, while transgenic plants displayed chlorotic symptoms. Furthermore, bacterial growth was measured to determine the bacterial proliferation inside plants, showing that transgenic plants exhibited significantly higher bacterial proliferation than that in wild-type plants at 3 days (**Figure 3E**). These results suggest that *ScRIPK* plays role in regulating drought tolerance and disease defense in *Arabidopsis*.

### 3.4 ScRIPK is an autophosphorylation protein kinase, adopting an active conformation

**Figure 4:**
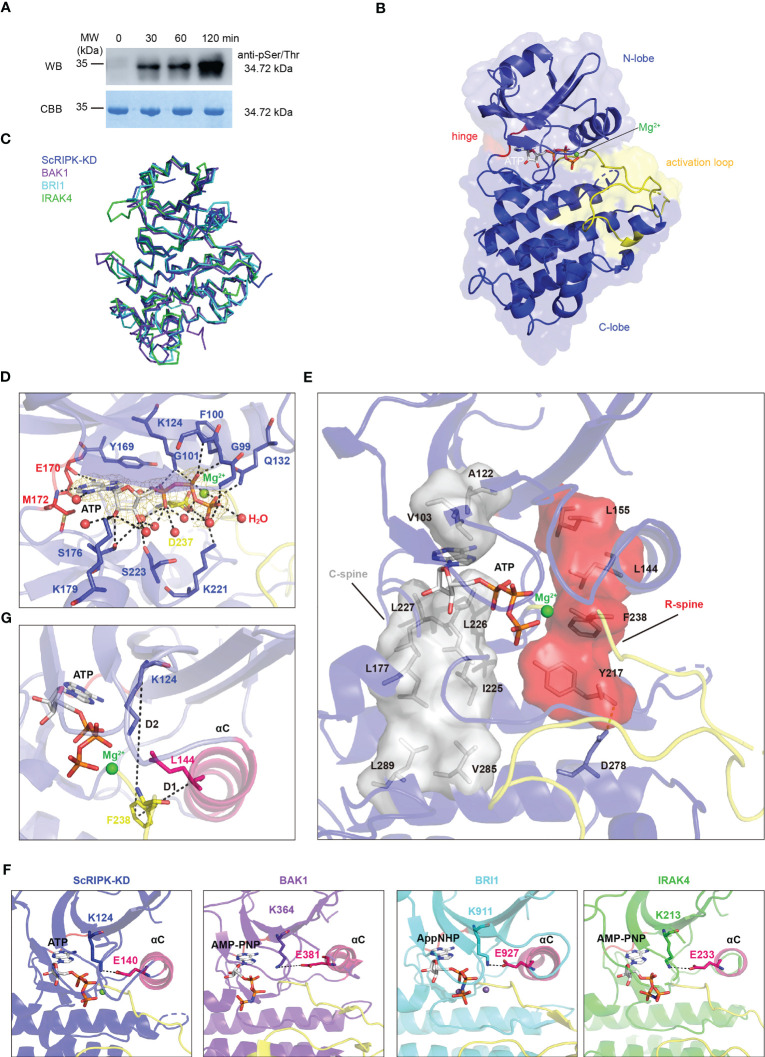
The autophosphorylation activity and overall structure of ScRIPK kinase domain complex with ATP. **(A)** Autophosphorylation of ScRIPK detected by western blot analysis using anti-Phospho-(Ser/Thr) antibody. The sample was collected at different time points with ATP and then separated in 12% SDS-PAGE, subsequently transferred to the PVDF membrane and immunoblotted with anti-Phospho-(Ser/Thr) antibody. MW represents molecular weight, and the molecular weight of ScRIPK-KD (34.72 kDa) was predicted by Expasy. **(B)** Structure of ScRIPK kinase domain (ScRIPK-KD) is shown in cartoon representation. The N-terminal lobe (N-lobe, 72–169 aa) is shown in slate blue, C-terminal lobe (C-lobe, 237–266 aa) is shown in tv_blue, hinge region (170–172 aa is shown in red, activation loop is shown in yellow, Mg^2+^ is shown in green, and ATP is shown in grey. **(C)** Ribbon structure comparison of ScRIPK kinase domain (ScRIPK-KD) with IRAK4, BAK1, and BRI1. **(D)** Detailed view of the ScRIPK-KD nucleotide binding sites. The 2*Fo-Fc* electron density map was contoured at 1.0σ, which was shown in gold mesh around ATP. The green and red spheres represent Mg^2+^ and water molecules, respectively. **(E)** Catalytic spine (C-spine) and regulatory spine (R-spine) formed in the ScRIPK KD-ATP complex. The C-spine is shown in grey, including the adenine base of ATP, V103, A122, L177, I255–L277, L289, and V285. The R-spine is shown in red, including L144, L155, F238, Y217, and D278. **(F)** Salt bridge (between Lys and Glu) in ScRIPK, IRAK4, BAK1, and BRI1. **(G)** Determined location of the Phe side chain of the DFG motif. D1 = distance (αC-Glu (+4)-Cα, DFG-Phe-Cζ), D2 = distance (β3-Lys-Cα, DFG-Phe-Cζ). All structure figures were prepared with PyMOL.

To determine the kinase activity of ScRIPK, an *in vitro* assay was performed with expressed ScRIPK kinase domain (72–378 aa, ScRIPK-KD) in *Escherichia coli*. Western blot analysis using a phosphor-Ser/Thr antibody confirmed the autophosphorylation of ScRIPK (**Figure 4A**). Most protein kinases phosphorylate and adopt active conformations. To explore the conformation and activation mechanism of ScRIPK, we determined the crystal structure of ScRIPK-KD using the molecular replacement method (**Table 1**). The solution compromises one ScRIPK-KD kinase domain per asymmetric unit. The ScRIPK-KD has a canonical bilobed structure architecture (**Figure 4B**). Like the typical structure of protein kinases, ScRIPK-KD contains a small N-lobe, composed of five β-sheets and an α-helix, and a large C-lobe, composed of six helices, an activation loop, and a catalytic loop (**Figures 4B, C**; **Supplementary Figure 1**). We performed three-dimensional homology searches using DALI (Holm et al., 2008), which indicated that ScRIPK-KD was closely related to the human interleukin receptor-associated kinase 4 (IRAK-4). A well-positioned ATP is one of the crucial features of a kinase catalytic machinery. In the ScRIPK-KD-ATP complex structure, Y169 and E170–M172 formed a deep cavity to clamp the adenine group of ATP and a magnesium ion bond with the catalytic base D237 and the β- and γ-phosphates of the nucleotide. Other residues, including G99–G101, K124, Q132, S176, K179, K221, and S223, may contribute to the stabilization of the nucleotide through hydrogen bonding (**Figure 4D**). The catalytic spine also facilitates the binding of ATP. The catalytic spine is completed by the adenine base of ATP, which binds to the V103 (from the beginning of the β2-strand), A122 (from the conserved Ala-Xxx-Lys of the β3-strand), and L226 (from the middle of the C-lobe β2-strand). I225 and L227 binds to L177 at the beginning of the αD-helix, and L177 binds to V285 and L289 in the αF-helix (**Figure 4E**). We also analyzed the ScRIPK-KD activation loopresidues that mediated the kinase functions. This activation loop contains three phosphorylation sites, namely, T250, S253, and T254, as indicated by the electron density (**Supplementary Figure 2A**).

**Table 1:** Data collection and structure refinement statistics.

	ScRIPK-KD-ATP	ScRIPK-KD^K124A^-ATP	ScRIPK-KD^S253A|T254A^-ADP
Data collection			
Beam line	SSRF beamline BL17U1	SSRF beamline BL17U1	SSRF beamline BL17U1
*a*, *b*, *c* (Å)	69.17, 72.35, 79.39	42.66, 82.35, 98.45	43.82, 84.01, 98.81
*α*, *β*, *γ* (°)	90.00, 90.00, 90.00	90.00, 90.00, 90.00	90.00, 90.00, 90.00
Space group	P21 21 21	P21 21 21	P21 21 21
Wavelength (Å)	0.979183	0.979183	0.979183
Resolution range (Å)	32.06–2.10 (2.15–2.10)	82.35–1.68 (1.72–1.68)	84.01–1.95 (2.00–1.95)
No. of unique/observed reflections	23842/305095	39500/511035	27392/341811
Completeness (%)	99.7	97.4	99.9
CC*_1/2_*	0.998	0.997	0.999
Average I/σ (*I*)	13.0 (1.7)	14.8	15.2
R*_merge_* (%)	0.138	0.103	0.100
Refinement			
No. of reflections used	23236	38953	26901
R*work* (%)	20.35	18.68	19.82
R*_free_* (%)	23.36	21.30	22.83
RMSD bond distance (Å)	0.007	0.006	0.007
RMSD bond angles (°)	0.962	0.902	0.967
Average B-value (Å^2^)			
Protein atoms	48.11	26.69	33.41
Ligands atoms	37.34	29.06	44.39
Ramachandran plot (%)			
Favored	96.97	98.88	99.27
Allowed	3.03	1.12	0.73
Outliers	0.00	0.00	0.00
PDB-ID	8HO6	8HOA	8HOD

The ScRIPK kinase domain adopted an active conformation with a salt-bridge formed between K124 and E140, similar to the active conformation of other kinase proteins, such as IRAK-4, BAK1, and BRI1 (**Figure 4F**). In addition, we determined the location of the DFG-Phe residue and calculated its distance from two conserved residues (K124 and L144, labeled as D2 and D1, respectively) in the N-terminal domain (**Figure 4G**; **Supplementary Table 2**), revealing that it belongs to the DFGin group. By analyzing the Ramachandran region annotation (A, B, L, and E) for the X, D, and F residues of the ScRIPK X-DFG motif and the DFG-Phe χ_1_ rotamer, we found that the ScRIPK-KD structure was in the BLAminus cluster (**Supplementary Table 3**). Furthermore, the regulatory spine (R-spine), which determines the positioning of the protein substrates so that catalysis occurs, was assembled in the ScRIPK-KD structure (**Figure 4F**). The R-spine contained residues from the activation segment and the αC-helix, which assembled by the L155 (from the beginning of the β4-strand), L144 (from the C-terminal end of the αC-helix), F238 (from the activation segment conserved DFG motif), along with H/YRD-Tyr217 of the catalytic loop. The backbone of Y217 was anchored to the αF-helix by a hydrogen bond to a conserved aspartate residue (D278). These findings show that ScRIPK is an autophosphorylating protein and adopts an active conformation.

### 3.5 The mutant of ScRIPK the nucleotide binding site or phosphorylation site adopts inactive conformation

**Figure 5:**
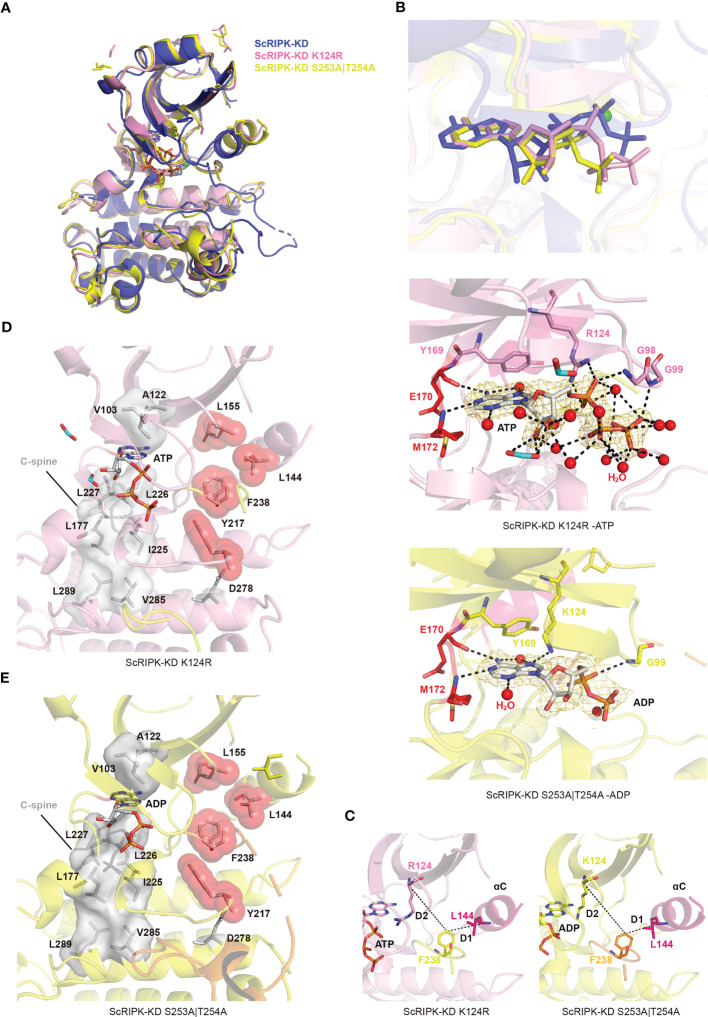
The overall structure of ScRIPK mutants. **(A)** The cartoon structure comparison of ScRIPK wild type and its mutants (ScRIPK K124R and ScRIPK S253A|T254A). ScRIPK wild type is shown in blue, ScRIPK K124R is shown in pink, and ScRIPK S253A|T254A is shown in dark yellow. **(B)** Comparison of the nucleotide position among ScRIPK wild type and its mutants, and the detailed view of ScRIPK mutants nucleotide binding sites. The 2*Fo-Fc* electron density map was contoured at 1.0σ, which is shown in gold mesh around ATP/ADP. **(C)** Determined the location of the Phe side chain of the DFG motif in ScRIPK K124R and ScRIPK S253A|T254A. **(D)** Catalytic spine was formed in the ScRIPK K124R-ATP complex. The C-spine is shown in grey, including the adenine base of ATP, V103, A122, L177, I255–L277, L289, and V285. **(E)** Catalytic spine was formed in the ScRIPK S253A|T254A -ADP complex. It is shown in grey, including the adenine base of ADP, V103, A122, L177, I255–L277, L289, and V285.

The kinase-dead mutation of FER K565R eliminates *in vitro* phosphorylation activity and causes reduced responses of *Arabidopsis* root to RALF1(Haruta et al., 2018). K124 forms a salt bridge with conserved E233, further stabilizing the nucleotide. In this study, the catalytic base K124–Arginine (K124R) mutation was performed and the mutant protein ScRIPK-KD K124R was purified and crystallized with ATP. The alignment of overall structure between wild type and K124R mutant protein showed no significant conformation changes (root mean square deviation (RMSD) is 1.090 Å comparing 220 corresponding C_α_ atoms) except for the C-helix and activation loop (**Figure 5A**). The C-helix was outward, away from the active center, and the activation loop had no electron density map. In addition, the position of ATP in protein structure of wild type and K124R mutant, and the crucial residues of ATP binding pocket were partially different (**Figure 5B**). In the ScRIPK-KD K124R mutant structure, Y169 and E170–M172 formed a deep cavity to clamp the adenine group of ATP. Other residues, including G98, G99, and K124, may contribute to the stabilization of the nucleotide through hydrogen bonds (**Figure 5B**). The catalytic spine was also completed by the adenine base of ATP like the wild type (**Figure 5C**). To explore the conformation of this mutant protein, we analyzed whether there was the salt bridge between R124 and E140 and the results showed that there was no salt bridge between them (**Figure 5D**). We also measured the distance of D1 and D2, and dihedral angles of the X-D-F residues, which revealed that the ScRIPK-KD K124R mutant was classified into DFGin group and BLAplus cluster (**Supplementary Tables 2**, **3**). Furthermore, the regulation spine was analyzed, which was not assembled (**Figure 5C**).

BRI1 T1049A and BAK1 T455A mutants lost nearly all kinase activity (Wang et al., 2005; Wang et al., 2008). The biological effect of mutating T446, T449 and T450 is less pronounced. However, T450 substitution restores growth while retaining complete flg22 insensitivity (Wang et al., 2008). We analyzed the electron density of the activation loop and found three phosphorylation sites, T250, S253, and T254 (**Supplementary Figure 2A**). According to the sequence alignment of ScRIPK, BRI1, and BAK1 (**Supplementary Figure 1**), we simultaneously mutated S253 and T254 in the ScRIPK protein, purified and crystallized the mutant protein, and analyzed its conformation. The C-helix of the ScRIPK-KD S253A|T254A with ADP complex was outward, away from the active center, similar to the ScRIPK-KD K124R mutant protein (**Figure 5A**). In the ScRIPK-KD S253A|T254A mutant structure, Y169 and E170–M172 formed a deep cavity to clamp the adenine group of ADP. Residues G99 and K124 may contribute to the stabilization of the nucleotides through hydrogen bonding (**Figure 5B**), and the C-spine was completed by the adenine base of ADP to stabilize the ADP (**Figure 5E**). To explore the conformation of this mutant protein, we analyzed the salt bridge between K124 and E140, and measured the distance of D1 and D2 and dihedral angles of the X-D-F residues. This revealed that there was no salt bridge between K124 and E140, and this mutant was classified into DFGin group and BLAplus cluster (**Figure 5C**; **Supplementary Figure 2C**; **Supplementary Tables 2**, **3**). In addition, the regulation spine was analyzed, which was also not assembled (**Figure 5E**). Therefore, the two mutant proteins were shown to adopt inactive conformation, suggesting that K124, S253, and T254 function in nucleotide binding and catalysis occurring.

### 3.6 ScRIPK interacts with RPM1-interacting protein 4

**Figure 6:**
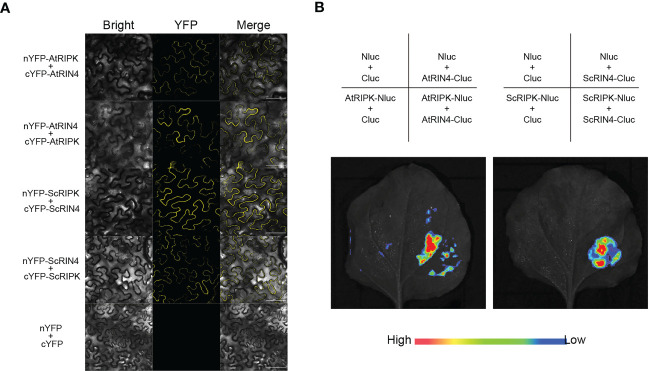
Isolation of the ScRIPK interaction protein ScRIN4. **(A)** BiFC analysis of the interaction between ScRIPK and ScRIN4 in *Nicotiana benthamiana* leaves. **(B)** Split-luciferases complementation assay showing the interaction between ScRIPK and ScRIN4 in *Nicotiana benthamiana* leaves. Infiltration with the empty vectors, AtRIPK and AtRIN4 were used as the negative control or positive control. Bars = 100 µm.

The receptor-like cytoplasmic kinase AtRIPK interacts with AtRIN4 and phosphorylates it, activating plant’s innate immune receptor (Liu et al., 2011). In the present study, we identified and cloned a protein in sugarcane (cv. ROC22) through homologous cloning. The deduced coding sequence contained a 702 bp open reading frame encoding a protein with 233 amino acid residues. Phylogenetic analysis revealed that the deduced protein was related to SbRIN4 (**Supplementary Figure 3A**). Therefore, we named this protein ScRIN4. Then we aligned the sequences of RIN4s from monocots and dicots to analyze the F/YTxxFxK motif surrounding the phosphorylation sites, which showed that ScRIN4 had a conserved motif similar to other RIN4 orthologs (**Supplementary Figure 3B**). To explore whether ScRIPK interacts with ScRIN4, as the homologs do in *Arabidopsis*, we used bimolecular fluorescence complementation (BiFC) and luciferase complementation imaging (LCI) assays. The results showed that ScRIPK did interact with ScRIN4 (**Figures 6A, B**; **Supplementary Figure 4**) and the results of the interaction between AtRIPK and AtRIN4 was used as the positive control.

## 4 Discussion

RLCKs are involved in various activities in plant growth and development, such as stress responses, which exist in many plants (Shiu et al., 2004; Vij et al., 2008; Fan et al., 2018). However, knowledge about the function of RLCKs in sugarcane is limited. Therefore, identifying and characterizing of genes that are related to coping with challenging environmental conditions are essential to developing new cultivars with better stress tolerance to ensure sustainable crop yields. In this study, ScRIPK, a member of the RLCK family in sugarcane, was isolated and functionally characterized. The obtained high-resolution crystal structure of ScRIPK kinase domain provides a reliable basis for the activation mechanism of kinase, and provides a target for genetic manipulation.

In rice, the loss of function of *OsGUDK* increases the sensitivity of seedlings to salinity, osmotic stress, and ABA treatment. At the vegetative stage, the survival rate of *osgudk* mutant lines is about half that of wild-type lines under drought treatment. (Ramegowda et al., 2014). ABA, salt, and drought stresses influenced the expression levels of *GsRLCK*, and the *Arabidopsis* lines that overexpress *GsRLCK* confer increased tolerance to salt and drought stresses, increasing the expression levels of genes which are related to stress responses (Sun et al., 2013). In our study, we identified a member of the RLCK VII subfamily, ScRIPK, which contained 11 conserved subdomains in its kinase domain and localized in the plasma membrane (**Figure 1**), similar to other RLCKs such as AtRIPK, AtBIK1, OsGUDK, and ZmBLK1 (Liu et al., 2011; Ramegowda et al., 2014; Li et al., 2021). Transcription levels of *ScRIPK* were influenced by PEG treatment (**Figure 2A**) and overexpression of *ScRIPK* in *Arabidopsis* increased the tolerance of seedlings to drought conditions (**Figures 3B, C**), which suggests that *ScRIPK* may be involved in drought stress signaling, similar to other RLCKs such as *OsGUDK* and *GsRLCK*. Protein kinases are important components of plant immunity, regulating plant responses to pathogens at multiple levels. An *Arabidopsis* RLCK from subfamily VII- *pbl13* T-DNA mutant lines exhibit increased resistance to *Pto* DC3000, and *AtPBL13* acts as a negative regulator of microbe-associated molecular pattern-triggered immunity (Lin et al., 2015). The *Arabidopsis* mutants *bik1* and *ripk* show strong resistance to *Plasmodiophora brassicae* and *Pto* DC3000, respectively (Liu et al., 2011; Chen et al., 2016). *ScRIPK* expression was also affected by pathogen infection (**Figure 2C**), with transgenic lines showing enhanced disease susceptibility (**Figures 3D, E**). These results indicate that *ScRIPK* may play an essential role in plant immunity and might function as a negative regulator of plant basal defense responses, similar to the function of genes like *AtRIPK* and *AtBIK1* in regulating defense responses.

Protein kinases regulate cellular signaling by phosphorylating their substrates in response to environment-specific stresses. Most protein kinases require phosphorylation to become active conformations (Yan et al., 2012; Bojar et al., 2014). For many kinases, activation requires phosphorylation of the activation segment, mediated by auto- or trans-phosphorylation (Nolen et al., 2004). Both OsGUDK and AtRIPK are autophosphorylating kinases, and the autophosphorylation activity of OsGUDK is Mg^2+^ dependent (Liu et al., 2011; Ramegowda et al., 2014). The present work determined the crystal structure of the ScRIPK KD-ATP complex to elucidate the activation mechanism. We found that ScRIPK is an autophosphorylating kinase that the activation loop of ScRIPK KD contained three phosphorylation sites (T250, S253, and T254), supported by electron density data (**Supplementary Figure 2**) and the western blot analysis (**Figure 4A**). The structure of this complex showed an active conformation, adopting αC-in and DFGin conformation and forming the salt bridge between K124 and E140 (**Figures 4F, G**; **Supplementary Tables 2**, **3**), which is a prerequisite for the formation of the active state, and corresponds to the αC-in conformation (Roskoski, 2015; Modi and Dunbrack, 2019). In this structure, ATP adopted a proper position: the adenine group of ATP bound to the conserved hydrophobic pocket, the nucleotide was stabilized through hydrogen bonds, α- and β-phosphates of the nucleotide directly interacted with K124, the salt bridge formed by K124 and E140 further stabilized the position of ATP, and the γ-phosphate was chelated with Mg^2+^, which in turn interacted with D237 of the DFG motif, so that the phosphate group was in a position suitable for catalysis (**Figure 4D**). In addition, the formation of the catalytic and regulatory spines (**Figure 4E**) facilitated the ATP binding and dictated the position of protein substrate so that catalysis occurred. The correct alignment of the regulatory spine is necessary for production of active kinases (Roskoski, 2015). Furthermore, the crystal structure of mutants ScRIPK K124R and ScRIPK S253A|T254A were determined and shown to adopt inactive conformations, with the αC-out conformation belonging to the BLAplus cluster in the DFGin group (**Figure 5**; **Supplementary Tables 2**, **3**). The R-spine was not assembled in the ScRIPK K124R structure or ScRIPK S253A|T254A structures (**Figures 5D, E**). There were incomplete or absent electron density maps for the activation loop of these two proteins and the T250 of ScRIPK S253A|T254A did not phosphorylate according to the electron density map (**Supplementary Figure 2B**). Therefore, we speculate that K124, S253, and T254 might be essential in stabilizing nucleotides and allowing catalysis to occur.

Many RLCKs localize to the plasma membrane *via* N-myriotoylation and/or a palmitoylation motif. Additionally, the plasma membrane localization increases interaction between RLCKs and other membrane proteins. AtRIN4, a plant immune regulator, has been known to target multiple bacterial effectors, such as AvrB, AvrPto, AvrRpm1, and AvrRpt2 (Mackey et al., 2002; Axtell and Staskawicz, 2003; Luo et al., 2009). RPM1, an NB-LRR immune receptor, recognizes the *Pseudomonas syringae* effectors AvrB and AvrRpm1 and recognizes AtRIN4 phosphorylation, leading to activation of effector-triggered immunity in *Arabidopsis* (Xu et al., 2017). AvrB-induced AtRIN4 phosphorylation depends on AtRIPK, which phosphorylates AtRIN4 at residues T21 and T166 of the conserved F/YTxxFxK motif (Liu et al., 2011). In the present study, we isolated the ScRIN4 and analyzed the amino acid sequences among ScRIN4 and its orthologs in other species (**Supplementary Figure 3**). The result showed that the F/YTxxFxK motif surrounding the phosphorylation site was conserved, but there was a slight difference in the final residue of the F/YTxxFxK motif (**Supplementary Figure 3B**), which may reflect a difference between monocots and dicots. Consistent with the interaction between AtRIPK and AtRIN4 in *Arabidopsis* (Ramegowda et al., 2014), ScRIPK interacted with ScRIN4 in sugarcane (**Figure 6**), suggesting that this interaction may be conserved in monocots and dicots. *AtRIN4* knockout and overexpression lines exhibit increased and decreased disease susceptibility to *Pst* DC3000 (Kim et al., 2005). These results suggest that ScRIPK might be involved in defense regulation by interacting with and phosphorylating ScRIN4.

Our findings provide new insights for studying of RLCKs, which might have potential applications in sugarcane molecular breeding and provide a structural basis for kinase activation mechanisms.

## Data availability statement

The datasets presented in this study can be found in online repositories. The names of the repository/repositories and accession number(s) can be found in the article/**Supplementary Material**.

## Author contributions

JF and ZC performed the experiments and analyzed the data. RH and CH participated in the experiments. JF wrote the manuscript. ZM guided proteins crystallization and data collection. MZ, WY, ZC, and BC revised the manuscript. JF, ZC, MZ, and WY designed the study. The authors have read and approved the final manuscript.
