# Supplementary figures and images for: Receptor-like cytoplasmic kinase ScRIPK in sugarcane regulates disease resistance and drought tolerance in Arabidopsis
Source: Front Plant Sci. 2023 May 25;14:1191449. doi: 10.3389/fpls.2023.1191449 (PMC10248867; doi:10.3389/fpls.2023.1191449)

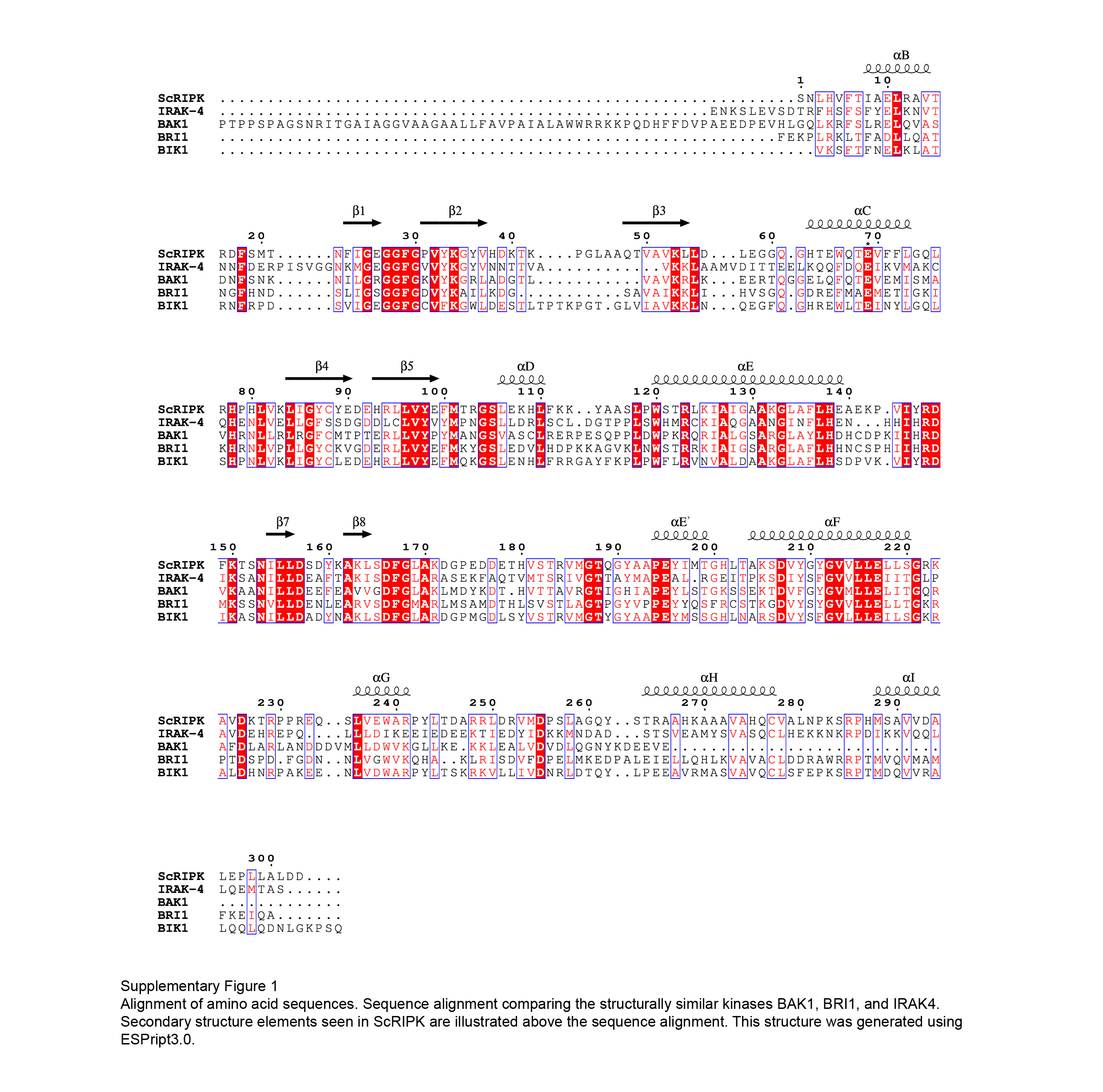

Supplement: Supplementary file 1 [file Image_1.jpeg]

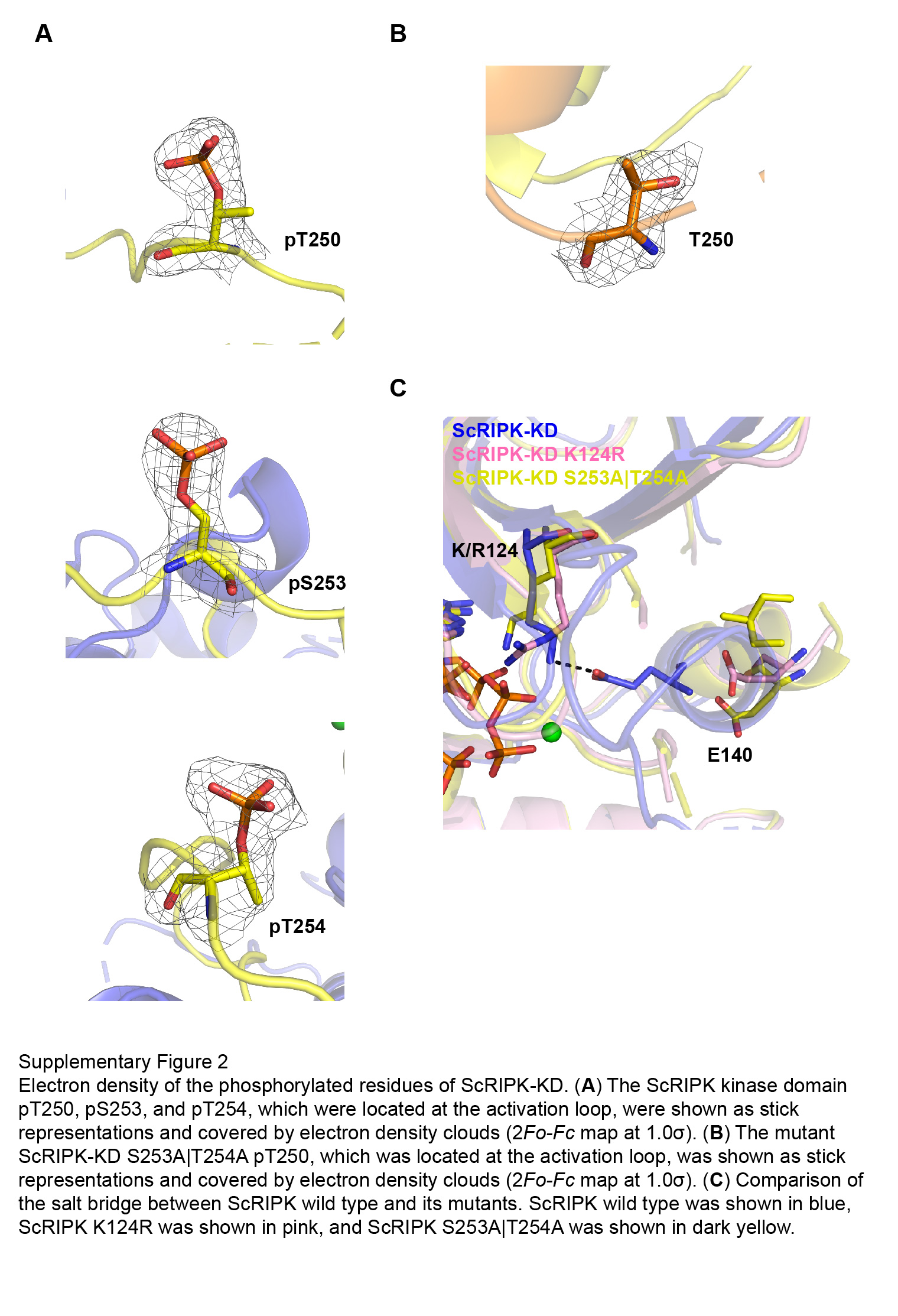

Supplement: Supplementary file 2 [file Image_2.jpeg]

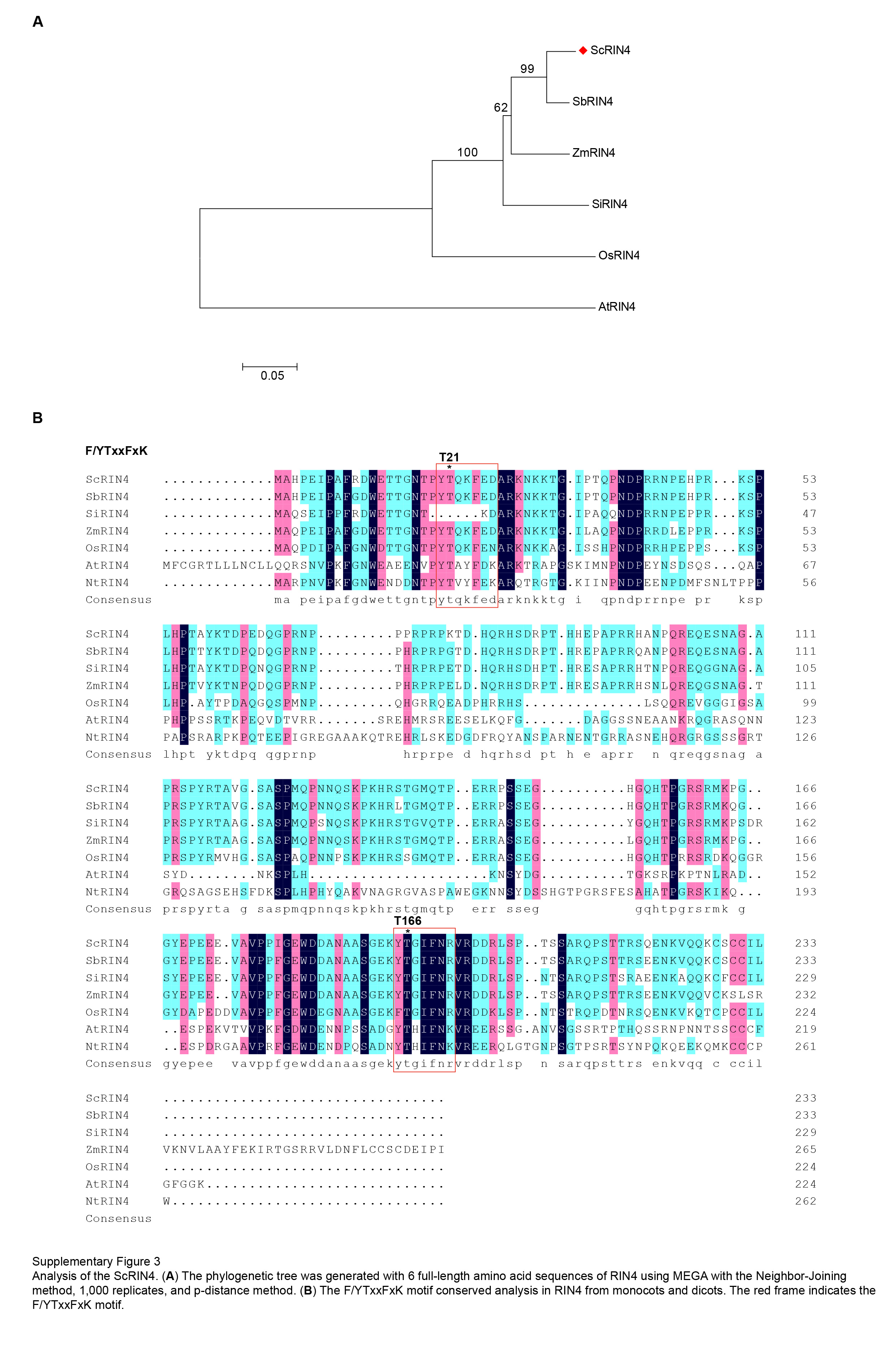

Supplement: Supplementary file 3 [file Image_3.jpeg]

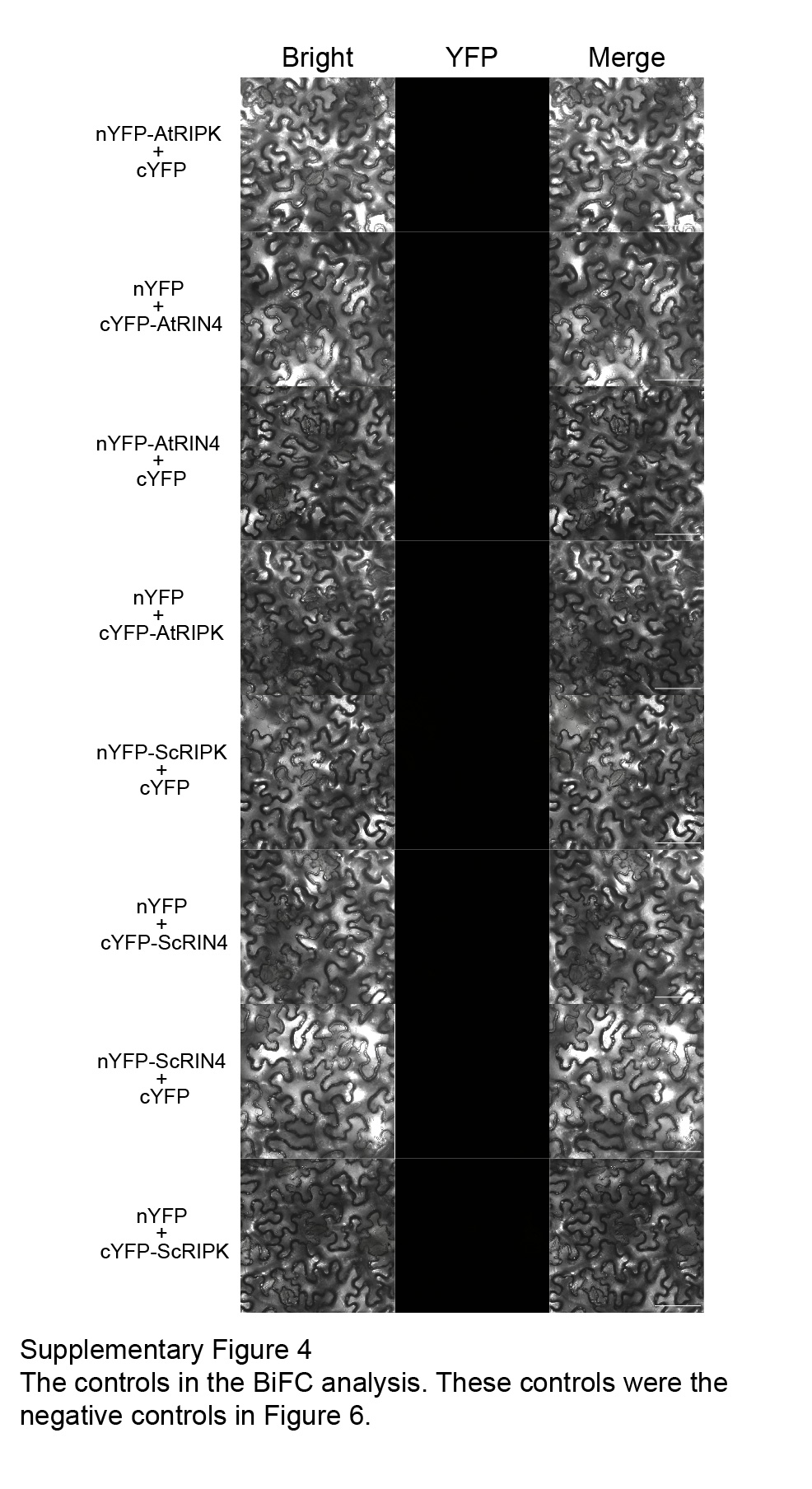

Supplement: Supplementary file 4 [file Image_4.jpeg]
