# Supplementary material for: Receptor-like cytoplasmic kinase ScRIPK in sugarcane regulates disease resistance and drought tolerance in Arabidopsis
Source: Front Plant Sci. 2023 May 25;14:1191449. doi: 10.3389/fpls.2023.1191449 (PMC10248867; doi:10.3389/fpls.2023.1191449)
Supplement: Supplementary file 5 [file Table_1.docx]

**Supplementary Table 1. Primers used in this study.**

| **Primer name** | **Sequence（5'- 3')** | **Purpose** |
| --- | --- | --- |
| ScRIPK-F | ATGCGTCCMGCCAAAGCC | Gene clone |
| ScRIPK-R | TCACTCCTCYGGCCGCAC |  |
| ScRIN4-F | GCTGAATTCCGCCTTGCAGATC |  |
| ScRIN4-R | ACAGGAAGAGTTACAAGATAGGGA |  |
| ScRIPK-F | CGAATGGCAGACGGAAGTGT | RT-qPCR |
| ScRIPK-R | GCCTCTCGTCATGAACTCGT |  |
| GADPH-F | CACGGCCACTGGAAGCA |  |
| GADPH-R | TCCTCAGGGTTCCTGATGCC |  |
| AtActin2-F | TCCCTCAGCACATTCCAGCAG |  |
| AtActin2-R | AACGATTCCTGGACCTGCCTCATC |  |
| ScRIPK-F | CTCGGTACCCGGGATCCATGGCGAGGCCGGGGTGG | BiFC |
| ScRIPK-R | TACGAGATCTGGTCGACCTCCTCTGGCCGCACGACCG |  |
| ScRIN4-F | GGCGGTACCCGGGATCCAATGGCGCACCCTGAAATTCC |  |
| ScRIN4-R | AAGCTCTGCAGGTCGACAAGTATGCAGCAAGAACATT |  |
| AtRIPK-F | CTCGGTACCCGGGATCCATGGCGGTGAAGAAGAAAGTTTC |  |
| AtRIPK-R | TACGAGATCTGGTCGACGTACCGTTCCCCACCTGC |  |
| AtRIN4-F | GGCGGTACCCGGGATCCAATGGCACGTTCGAATGTACC |  |
| AtRIN4-R | AAGCTCTGCAGGTCGACTTTTCCTCCAAAGCCAAAG |  |
| ScRIPK-F | CGTCCCGGGGCGGTACCATGGCGAGGCCGGGGTGG | LUC |
| ScRIPK-R | AAGCTCTGCAGGTCGACCTCCTCTGGCCGCACGACCG |  |
| ScRIN4-F | GGACGAGCTCGGTACCCATGGCGCACCCTGAAATTCC |  |
| ScRIN4-R | TACGAGATCTGGTCGACAAGTATGCAGCAAGAACATT |  |
| AtRIPK-F | CGTCCCGGGGCGGTACCATGGCGGTGAAGAAGAAAGTTTC |  |
| AtRIPK-R | AAGCTCTGCAGGTCGACGTACCGTTCCCCACCTGCCT |  |
| AtRIN4-F | GGACGAGCTCGGTACCCATGGCACGTTCGAATGTACC |  |
| AtRIN4-R | TACGAGATCTGGTCGACTTTTCCTCCAAAGCCAAAGCA |  |
| ScRIPK-F | CCACAGCCAGGGATCCCTGGTTCCGCGTGGCTCCTCCAACCTCCACGTCTTCACCAT | Protein expression |
| ScRIPK-R | CTTTACCAGACTCGAGTCAGTCGTCGAGCGCGAGGAGC |  |
| ScRIPK-R1 | CGTCCCCATGACTCGCGCCGCCACGTGCGTCTCGTC | Mutation (S253A\|T254A) |
| ScRIPK-F2 | GACGAGACGCACGTGGCGGCGCGAGTCATGGGGACG |  |
| ScRIPK-R1 | CCTCGAGGTCGAGCAGCCTGACGGCGACGGTCTGC | Mutation (K124R) |
| ScRIPK-F2 | GCAGACCGTCGCCGTCAGGCTGCTCGACCTCGAGG |  |
