# Supplementary material for: Receptor-like cytoplasmic kinase ScRIPK in sugarcane regulates disease resistance and drought tolerance in Arabidopsis
Source: Front Plant Sci. 2023 May 25;14:1191449. doi: 10.3389/fpls.2023.1191449 (PMC10248867; doi:10.3389/fpls.2023.1191449)
Supplement: Supplementary file 6 [file Table_2.docx]

**Supplementary Table 2. Distance (D1 and D2) used to identify the location of the Phe side chain of the DFG motif.**

|  | **D1^a^** | **D2^b^** | **Group** |
| --- | --- | --- | --- |
| ScRIPK-KD-WT | 8.0 | 15.6 | DFGin |
| ScRIPK-KD-K124R | 6.1 | 12.6 | DFGin |
| ScRIPK-KD-S253A\|T254A | 5.8 | 13.4 | DFGin |

^a^D1 = distance (αC-Glu (+4)-Cα, DFG-Phe-Cζ).

^b^D2 = distance (β3-Lys-Cα, DFG-Phe-Cζ).
