# Supplementary material for: Receptor-like cytoplasmic kinase ScRIPK in sugarcane regulates disease resistance and drought tolerance in Arabidopsis
Source: Front Plant Sci. 2023 May 25;14:1191449. doi: 10.3389/fpls.2023.1191449 (PMC10248867; doi:10.3389/fpls.2023.1191449)
Supplement: Supplementary file 7 [file Table_3.docx]

**Supplementary Table 3. Determination of the DFG-Phe side chain position by measuring the dihedral angles^a^.**

|  | **X-DFG** | | **DFG-Asp** | | **DFG-Phe** | | **DFG-Phe** | **Cluster** |
| --- | --- | --- | --- | --- | --- | --- | --- | --- |
|  | PHI | PSI | PHI | PSI | PHI | PSI | χ_1_ rotamer |  |
| ScRIPK-KD-WT | -138.8 | 170.9 | 63.5 | 67 | -82.4 | 42.2 | -80.7 | BLAminus |
| ScRIPK-KD-K124R | -115.0 | 161.7 | 65.8 | 20.8 | -71.7 | 141.7 | 50.9 | BLAplus |
| ScRIPK-KD-S253A\|T254A | -121.8 | 161.3 | 63.8 | 18.5 | -72.7 | 142.4 | 54.3 | BLAplus |

^a^The dihedral angles contained X-DFG (X represent the residue before the DFG motif), DFG-Asp, DFG-Phe, and the χ_1_ of DFG-Phe.
